# Supplementary material for: Hominin-specific regulatory elements selectively emerged in oligodendrocytes and are disrupted in autism patients
Source: Nat Commun. 2020 Jan 16;11:301. doi: 10.1038/s41467-019-14269-w (PMC6965079; doi:10.1038/s41467-019-14269-w)
Supplement: Supplementary file 1 — Supplementary Information [file 41467_2019_14269_MOESM1_ESM.pdf]

Supplementary Information

"Hominin-specific regulatory elements selectively emerged in oligodendrocytes and are disrupted in autism patients"

Castelijns et al.

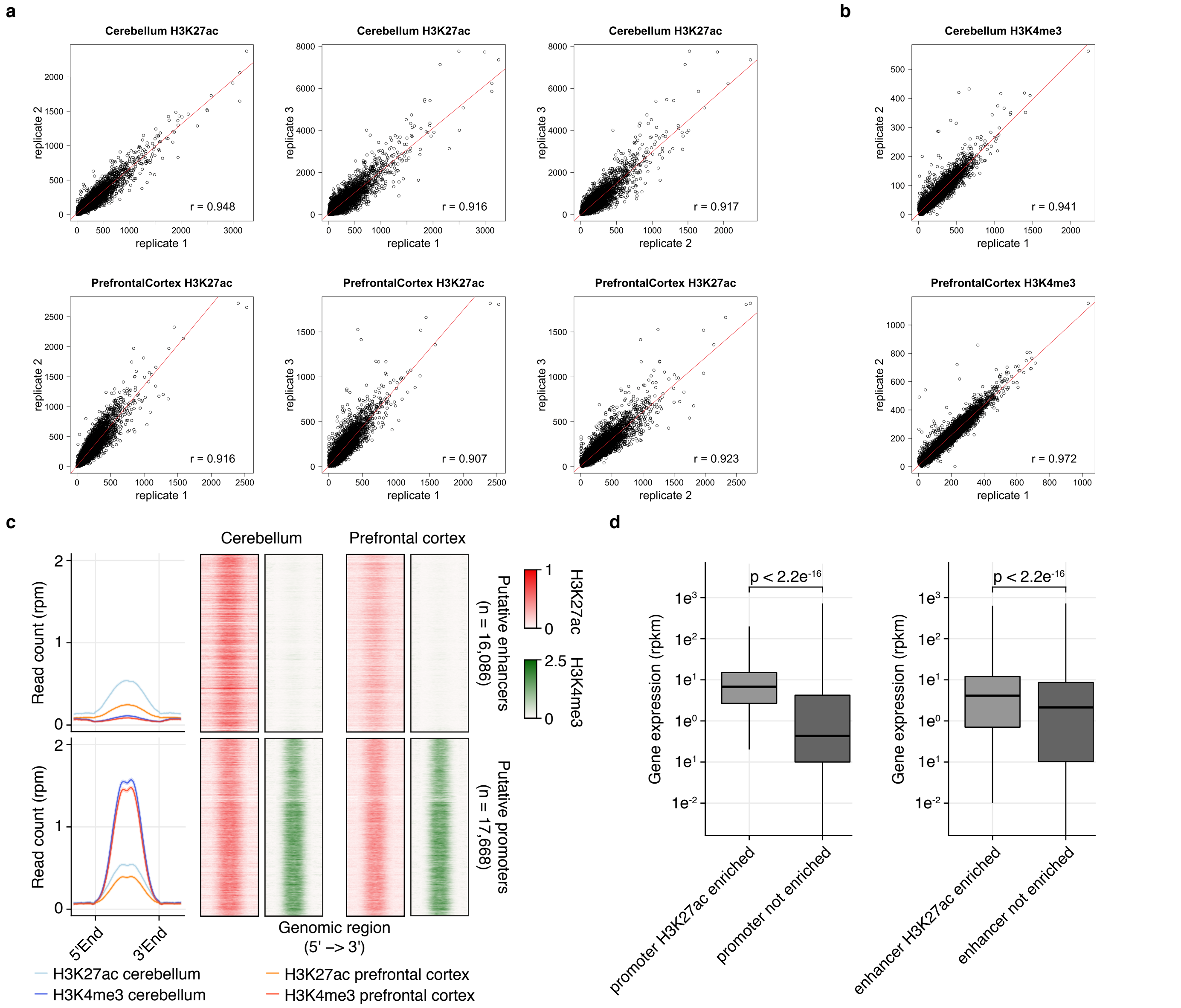

Supplementary Fig. 1 Annotation of marmoset regulatory elements. a, scatterplots showing the correlation of H3K27ac enrichment for all annotated marmoset regulatory elements between the replicate samples. Red line depictes linear regression through the samples, Pearson correlation between the samples is shown in the right bottom corner. **b,** similar as shown in **a** for H3K4me3 enrichment. **c,** metaplot analysis showing average enrichment profiles across scaled enriched regions (left panels). Colored lines represent average ChIP-seq enrichment for indicated sample, shaded area represents standard error of the mean. Heatmaps showing the H3K27ac (red) and H3K4me3 (green) enrichment in cerebellum and prefrontal cortex for putative enhancers and putative promoters across 5' to 3' scaled enriched regions. **d,** box plots showing normalized gene expression values in marmoset brain for genes with a H3K27ac enriched promoter or near a H3K27ac enriched enhancer compared to genes not linked to H3K27ac enrichment, outliers not shown. Dissimilarities between distributions were calculated using a Student's t-test. Bottom and top of the box plots are the first and third quartile. The line within the boxes represents the median and whiskers denote interval within 1.5x the interquartile range from the median.

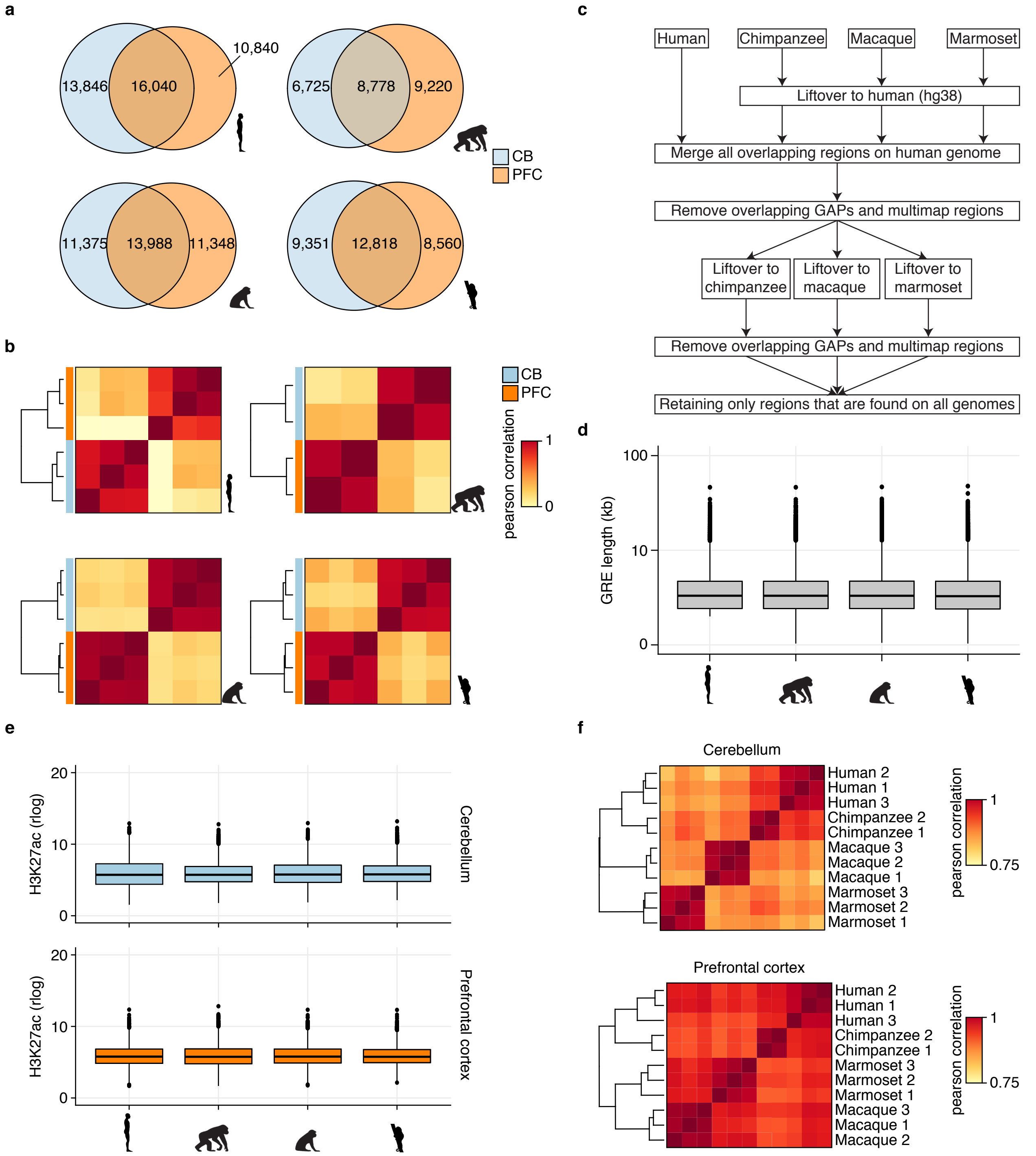

Supplementary Fig. 2 Integration of regulatory information across primate genomes. a, Venn diagram showing the overlap of regulatory elements identified in cerebellum (CB; blue) and prefrontal cortex (PFC; orange) for the different primate species as indicated. **b,** hierarchical clustering of brain samples for each primate species, based on regulatory elements identified in the separate species. Correlation map colors indicate Pearson correlation coefficients between the samples. Side bars are color-coded as in **a**. Pearson distances are represented by the tree on the left. **c,** schematic overview of the liftover pipeline used to identify GREs with ortholgous sequences on all primate genomes. **d,** box plots showing the length distribution of identified primate GREs per primate species. **e,** box plots showing the normalized H3K27ac enrichment of all identified primate GREs in cerebellum and prefrontal cortex for all primate species. Colors indicate tissue of origin as in **a**. **f,** hierarchical clustering of primate samples based on all identified primate GREs for cerebellum and prefrontal cortex. Heatmaps colors represent the Pearson correlation coefficients between samples. Pearson distances are represented by the tree on the left. Bottom and top of all box plots are the first and third quartile. The line within the boxes represents the median and whiskers denote interval within 1.5x the interquartile range from the median, outliers are depicted as points. Source data are provided as a Source Data file.

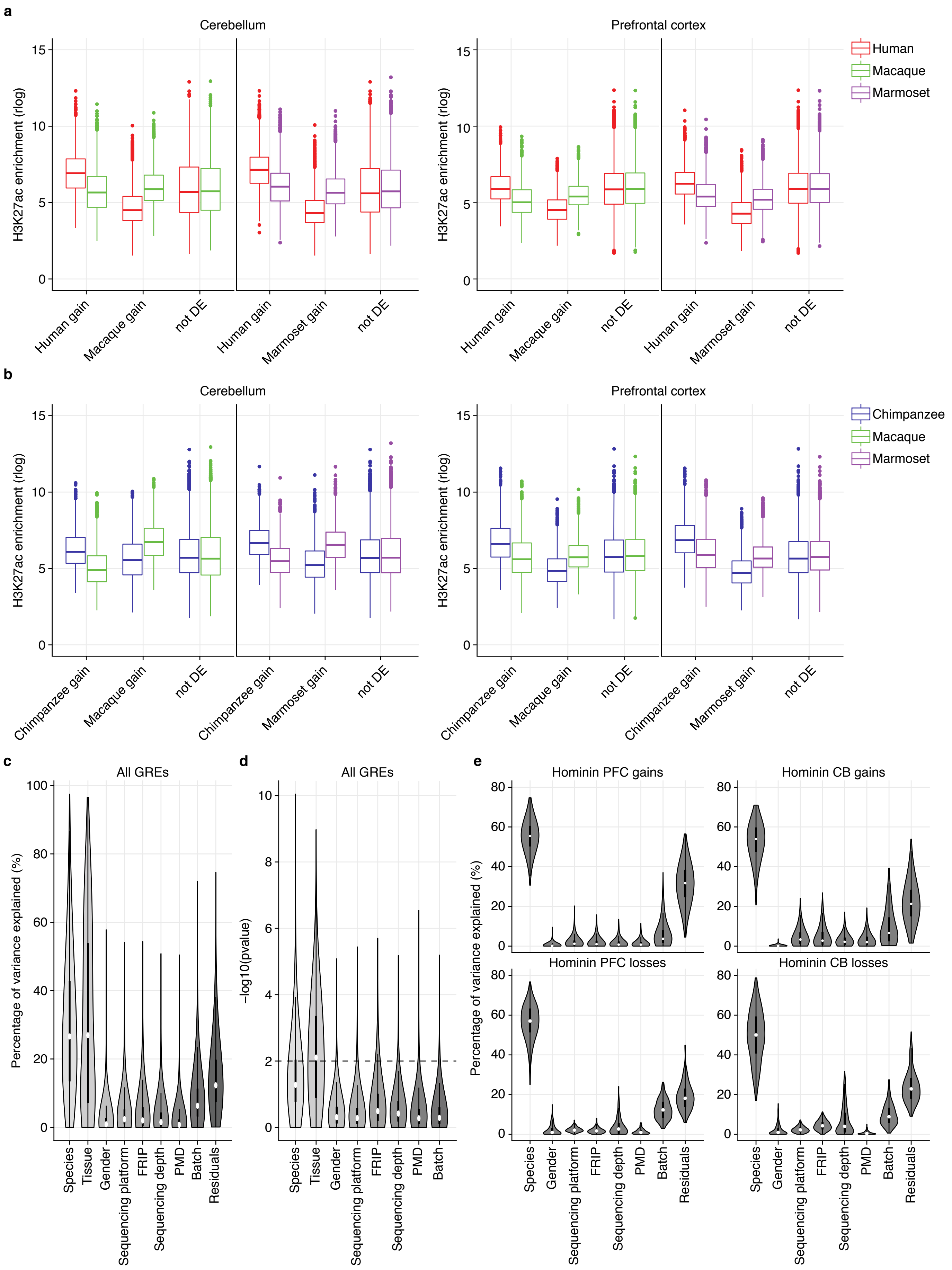

Supplementary Fig. 3 Differentially enrichment analysis in PFC and CB. a, box plots showing the average normalized H3K27ac enrichment per tissue as indicated for elements defined as human or monkey (macaque or marmoset) specific based on DESeq2 analysis. Colors indicate species (red; human, green; macaque, purple; marmoset). **b,** as in **a** using chimpanzee instead of human to compare to the monkey species. Colors indicated species (blue; chimpanzee, green; macaque, purple; marmoset). Bottom and top of the box plots are the first and third quartile. The line within the boxes represents the median and whiskers denote interval within 1.5x the interquartile range from the median, outliers are depicted as points. **c,** violin plots showing contribution of multiple covariates on H3K27ac enrichment assessed by a multivariate linear regression using ANOVA on all GREs across the primate samples. FRIP: fraction of reads in peaks, PMD: postmortem delay. **d,** similar as in **c** but showing the significance per covariate. Dashed line indicates the significance threshold $p < 0.01$. **e,** similar is in **c** but for hominin-specific changes in prefrontal cortex and cerebellum.

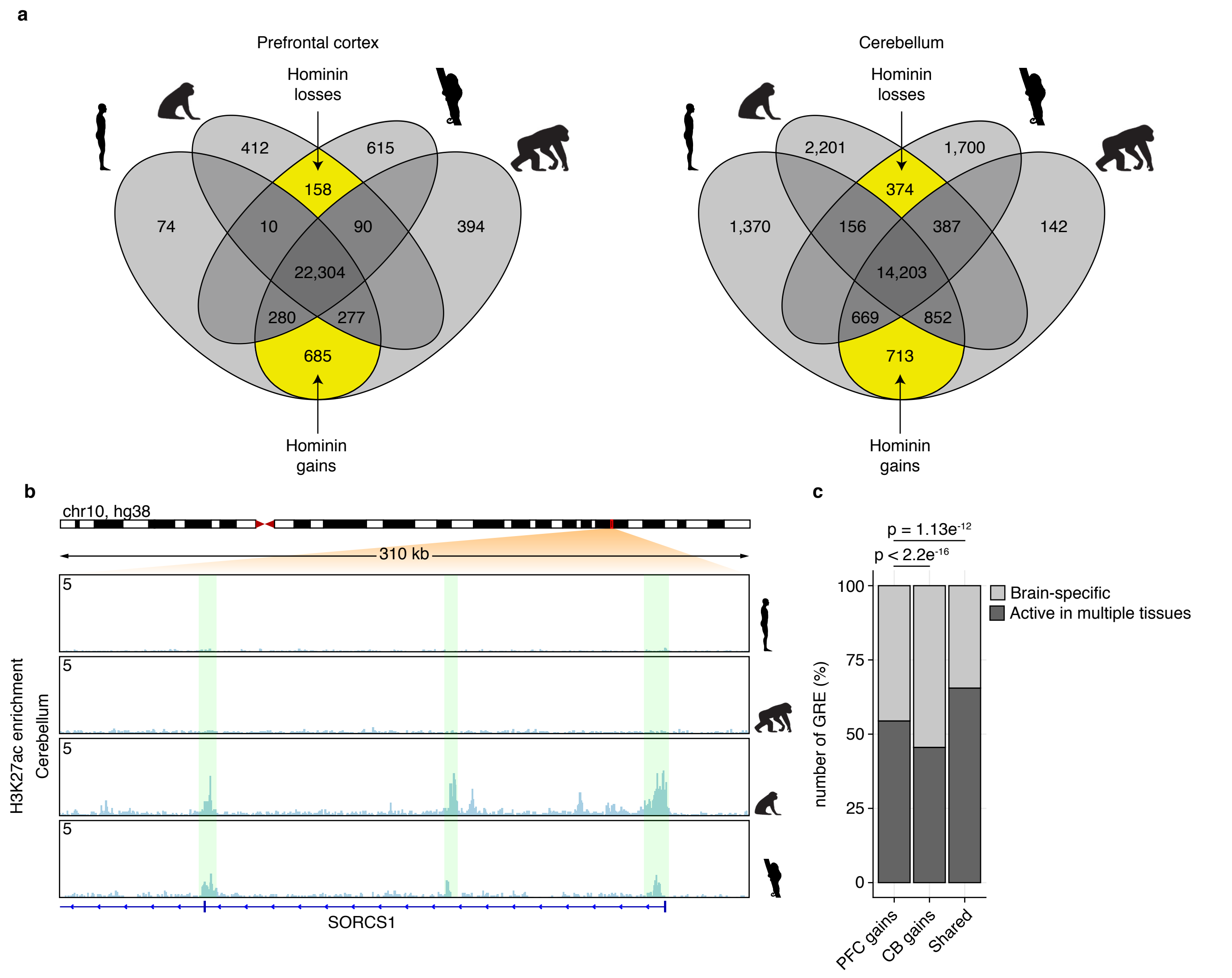

Supplementary Fig. 4 Identification of hominin-specific regulatory changes in PFC and CB. a, venn diagrams showing hominin-specific regulatory elements (highlighted in yellow) and species-specific gains and losses, as characterized based on differentially enrichment by DESeq2, for prefrontal cortex (left) and cerebellum (right). Numbers indicate the amount of regions in each catagory. **b,** ChIP-seq tracks showing rpm normalized H3K27ac enrichment across a 310kb region containing the SORCS1 gene in human cerebellum for the different species as indicated. The region harbours hominin-specific losses (highlighted in green). **c,** specificity of hominin-specific gains in other human tissues compared to regions that show no differentially enrichment between the primates. Difference in their frequency was calculated using a Fisher's exact test. Source data are provided as a Source Data file.

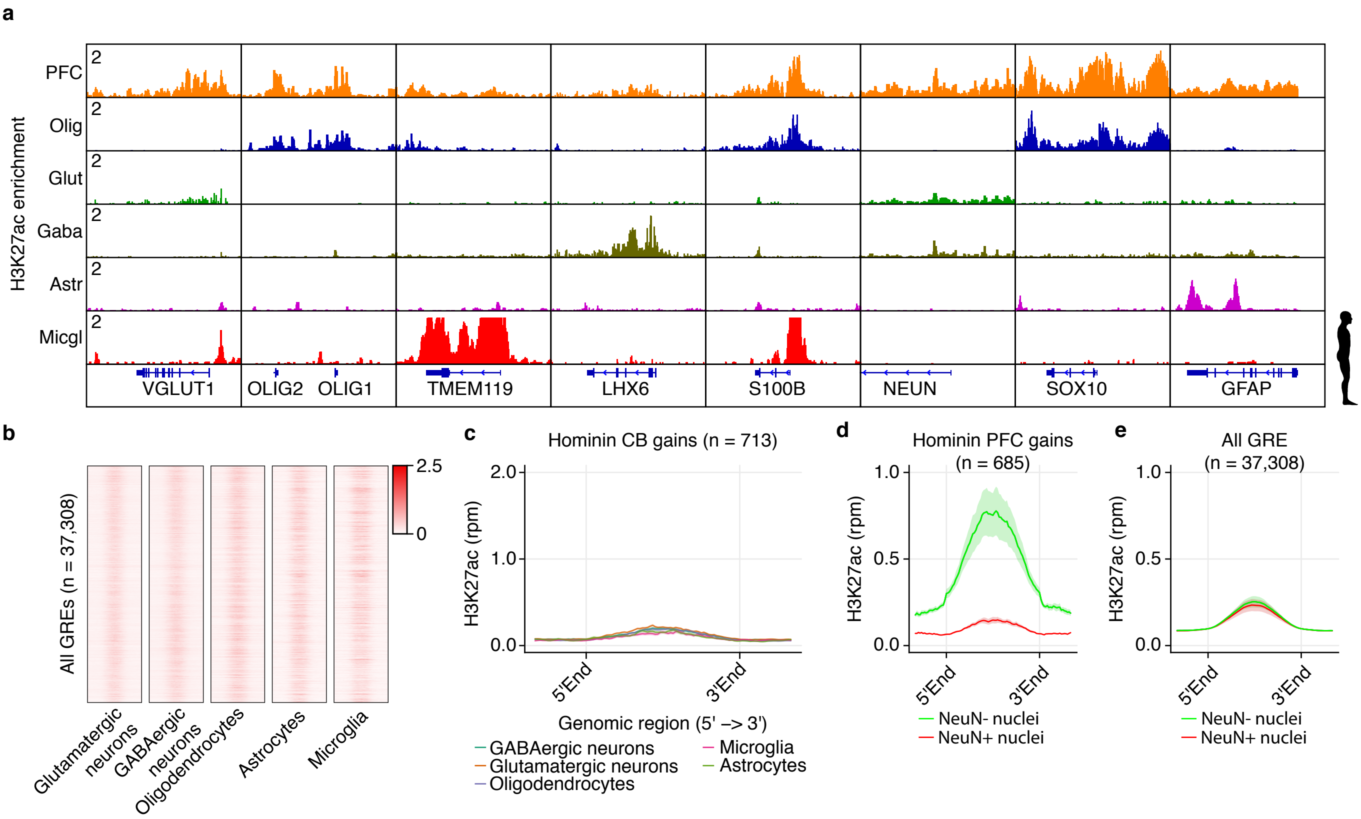

Supplementary Fig. 5 Analysis of hominin-specific regulatory elements at cell type resolution. a, ChIP-seq tracks showing rpm normalized H3K27ac enrichment across several known marker genes specific for neuronal and glial cell types in the human brain. Markers are VGLUT1 for glutamatergic neurons (Glut), Olig 1, 2 and Sox10 for oligodendrocytes (Olig), LHX6 for GABAergic neurons (Gaba), NEUN for neurons, S100B for Glia, TMEM119 for microglia (Micgl) and GFAP for astrocytes (Astr). **b,** heatmap showing H3K27ac enrichment across scaled regions for all GREs identified in PFC and CB across primates and analysed in the different neuronal and glial cell types as indicated. Heatmap colors indicated H3K27ac enrichment (rpm). **c,** metaplot analysis showing the average H3K27ac enrichment profile in different cell types for hominin-specific CB gains. **d,** metaplot analysis showing the average rpm-normalized H3K27ac enrichment profile of three NeuN- (green) and three NeuN+ (red) sorted nuclei samples on hominin-specific PFC gains. Shaded area depicts the standard deviation from the mean. **e,** Metaplot as shown in **d** but for all GREs.

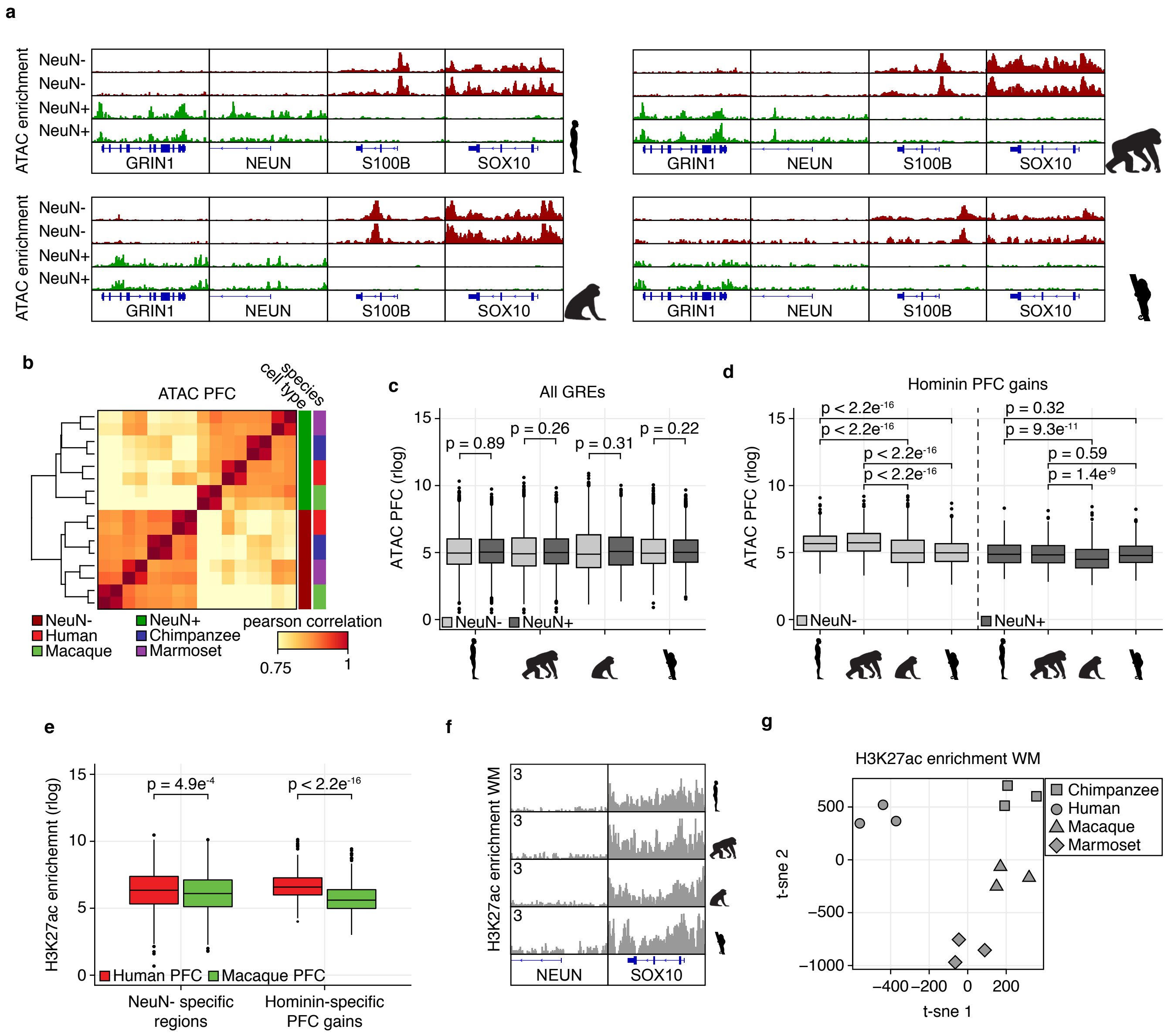

Supplementary Fig. 6 Hominin-specific gains emerged in non-neuronal cells. a, ATAC-seq tracks showing the rpm normalized ATAC signal for NeuN-negative sorted nuclei (green) and NeuN-positive sorted nuclei (red) across several known marker genes specific for neuronal cells (NeuN & vGlut1) and glial cells (S100B and Sox10) in four primate species as indicated. **b,** hierarchical clustering of NeuN sorted nuclei samples from prefrontal cortex of different primate species. Correlation map colors indicate Pearson correlation coefficients between samples. Side bars are color-coded based on sample origin and NeuN signal. Pearson distances are represented by the tree on the left. **c,** box plots showing normalized ATAC enrichment for all primate GREs on nuclei sorted for NeuN in different primate species. Dissimilarities between the distributions were calculated using a Student's t-test. **d,** box plots showing normalized ATAC enrichment in nuclei sorted for NeuN from different primate species as indicated on hominin-specific PFC gains. Dissimilarities between the distributions were calculated using a Student's t-test. **e,** box plots showing normalized H3K27ac enrichment in the prefrontal cortex of human and macaque for NeuN- specific regions and hominin-specific PFC gains. Dissimilarities between the distributions were calculated using a Student's t-test. **f,** ChIP-seq track showing the rpm normalized H3K27ac enrichment across NeuN and Sox10 loci for white matter tissue of the different primates as indicated. **g,** t-Distributed Stochastic Neighbor Embedding (t-sne) analysis of H3K27ac enriched GREs in white matter tissue with orthologs on all four primate genomes (n = 23,437). Axes indicate semantic space. Bottom and top of all box plots are the first and third quartile. The line within the boxes represents the median and whiskers denote interval within 1.5x the interquartile range from the median, outliers are depicted as points. Source data are provided as a Source Data file.

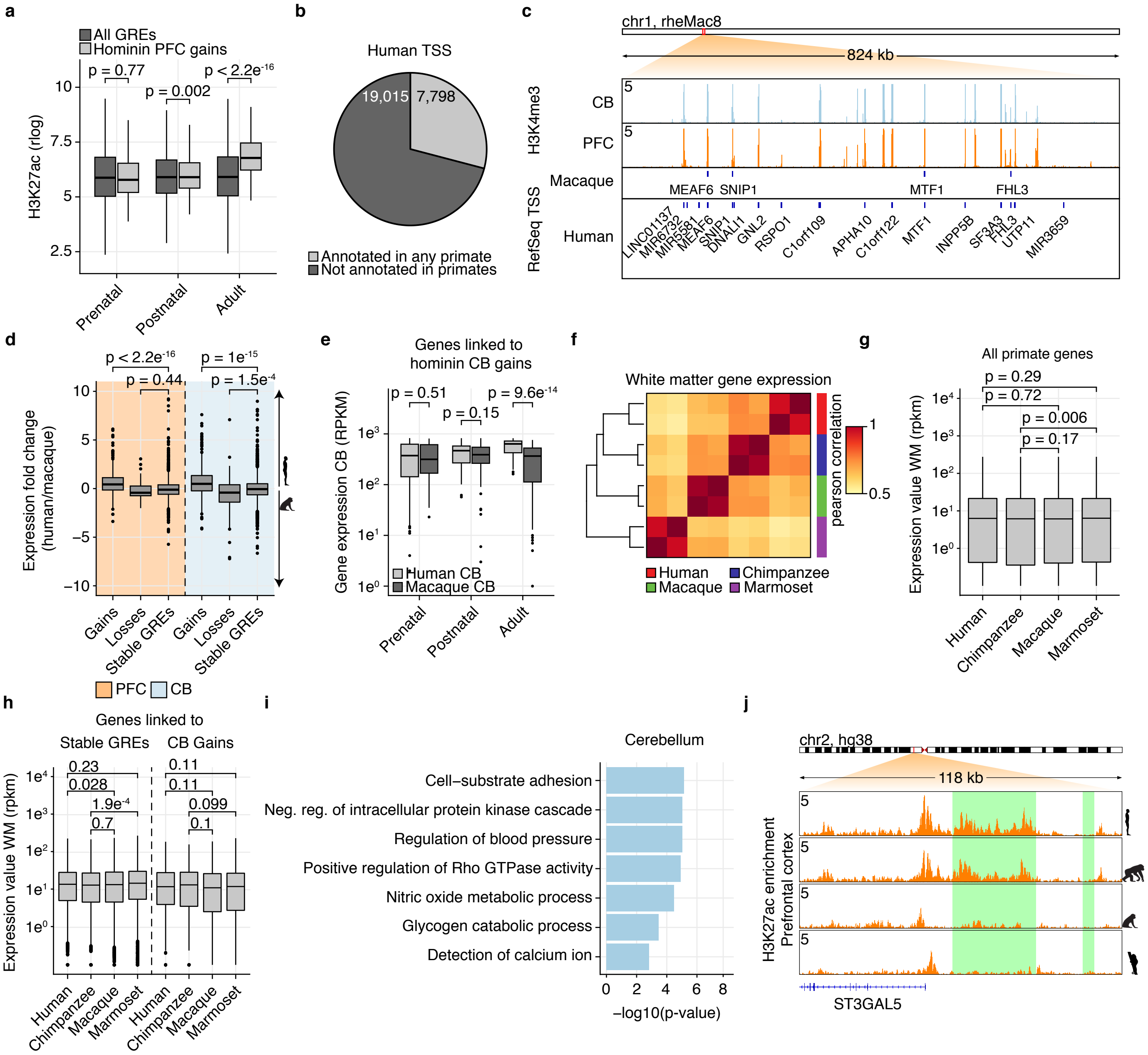

Supplementary Fig. 7 Hominin-specific GREs link to postnatally active genes. a, box plots showing normalized H3K27ac enrichment per developmental stage for all here identified GREs and hominin-specific PFC gains. Dissimilarities between the distributions were calculated using a Student's t-test. **b,** pie chart showing the overlap in gene annotation of known human TSS on primate species. **c,** ChIP-seq tracks showing rpm normalized H3K4me3 enrichment across a 824kb region for macaque cerebellum and prefrontal cortex. UCSC-defined transcription start sites (RefSeq TSS) for macaque and human are depicted underneath. **d,** box plot showing the log-fold differences in gene expression values between human and macaque for genes linked to hominin-specific changes and stable GREs in the prefrontal cortex and cerebellum. Dissimilarities between distributions were calculated using a Student's t-test. **e,** box plots showing normalized gene expression values in cerebellum of genes linked to hominin-specific CB gains across developmental time, for both human and rhesus macaque. Dissimilarities between distributions were calculated using a Student's t-test. **f,** hierarchical clustering of RNA-seq samples from white matter tissue of different primates as indicated by the colored side bar. Correlation map colors indicate Pearson correlation coefficients between samples. Pearson distances are represented by the tree on the left. **g,** box plots showing normalized gene expression values in white matter of different primate species for all genes annotated on the primate genome. Dissimilarities between distributions were calculated using a Student's t-test. **h,** box plots as in **g** but for genes linked to hominin-specific CB gains and stable GREs. Dissimilarities between distributions were calculated using a Student's t-test. **i,** gene-onthology analysis using GREAT for genes near hominin-specific CB changes. P-values represent significance of enrichment for stated biological process. **j,** ChIP-seq tracks showing rpm normalized H3K27ac enrichment in prefrontal cortex samples for different primate species as indicated in a 118kb region surrounding the ST3GAL5 gene. Hominin-specific PFC gains are highlighted in green. Bottom and top of the box plots are the first and third quartile. The line within the boxes represents the median and whiskers denote interval within 1.5x the interquartile range from the median. Source data are provided as a Source Data file.

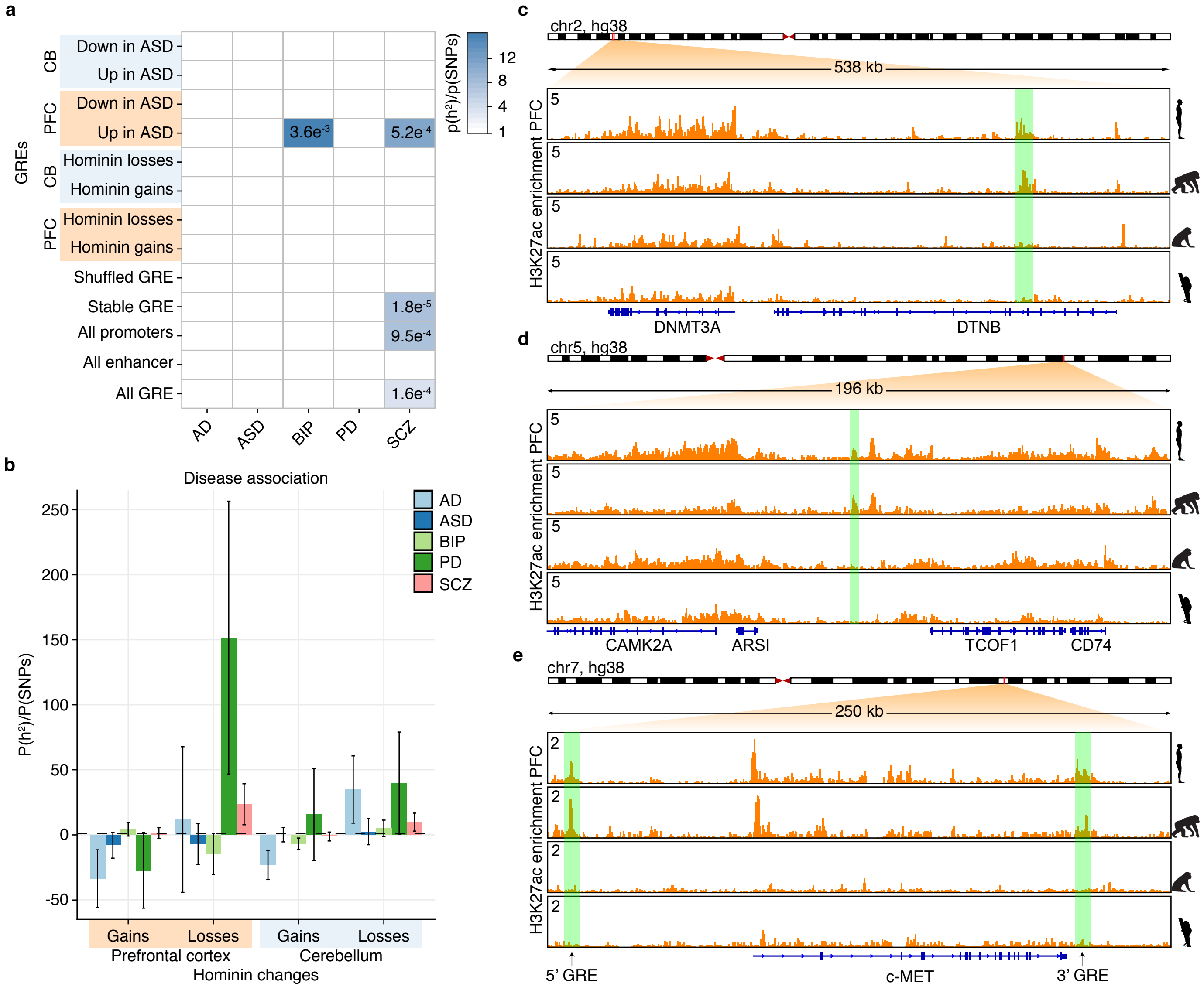

Supplementary Fig. 8 Hominin-specific gains are associated with neurological disease. a, heritability enrichment estimates for selected GREs and neurological traits using stratified LD score regression. Heatmap color indicate the proportion of heritability divided by the proportion of SNPs per selected set of GREs and are represented with associated p-value if $p < 0.01$. AD: Alzheimer's Disease, ASD: autism spectrum disorder, BIP: bipolar disorder, PD: Parkinson's disease, SCZ: schizophrenia. **b,** bar plots showing enrichment estimate for hominin-specific changes across different neurological traits. Error bars represent standard error around the estimate of enrichment. AD: Alzheimer's Disease, ASD: autism spectrum disorder, BIP: bipolar disorder, PD: Parkinson's disease, SCZ: schizophrenia. **c,** ChIP-seq track showing rpm normalized H3K27ac enrichment across a 538 kb region containing the DNMT3A gene in prefrontal cortex for four primate species as indicated. Hominin-specific PFC gain highlighted in green. **d,** ChIP-seq track showing rpm normalized H3K27ac enrichment across a 195 kb region containing the CAMK2A gene in prefrontal cortex for four primate species as indicated. Hominin-specific PFC gain highlighted in green. **e,** ChIP-seq track showing rpm normalized H3K27ac enrichment across a 250kb region containing the c-MET gene in prefrontal cortex for four primate species as indicated. Hominin-specific PFC gains are highlighted in green. Source data are provided as a Source Data file.

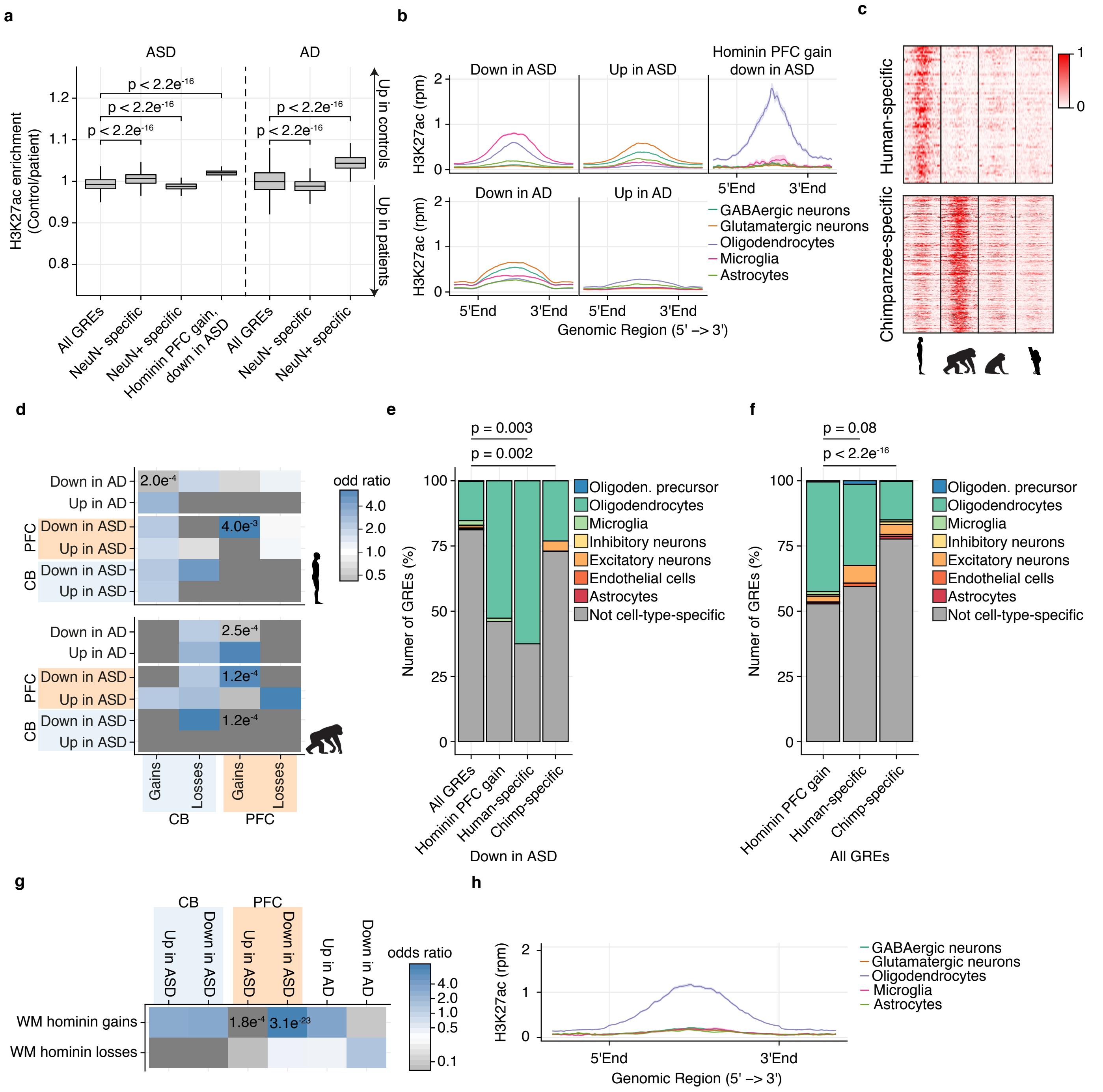

Supplementary Fig. 9 Cell type specificity for species-specific GREs deregulated in ASD. a, box plots showing the log-fold differences in normalized H3K27ac enrichment between ASD and AD patients and healthy control brains for all identifed GREs, regions either NeuN- or NeuN+ specific and regions identified as hominin-specific PFC gains deregulated in ASD. Dissimilarity between the distributions was calculated using a Student's t-test. Bottom and top of all box plots are the first and third quartile. The line within the boxes represents the median and whiskers denote interval within 1.5x the interquartile range from the median. **b,** metaplot analysis showing the average H3K27ac enrichment profile in different cell types for regions differentially enriched in either autism spectrum disorder (ASD) or Alzheimer's disease (AD) brains compared to control samples and regions identified as hominin-specific PFC gain deregulated in ASD. **c,** heatmap showing rpm normalized H3K27ac enrichment in the PFC of different primate species as indicated, on scaled human and chimpanzee-specific PFC regulatory elements. **d,** heatmap showing the enrichment of disease associated GREs measured to be deregulated in autism spectrum disorder and Alzheimer's disease patient brains in correlation to human and chimpanzee-specific changes found in cerebellum (CB) and prefrontal cortex (PFC). Color coding indicates odds ratio of enrichment compared to all identified GRE. P-values were determined using a Fisher's exact test and represented in the heatmap if $p < 0.01$. **e,** barplot showing cell-type specificity, based on single cell ATAC, of regions identified to be down regulated in autism patients brains. Shown are: all ASD down regions and those that overlap either hominin, human or chimpanzee-specific gains. Difference in oligodendrocyte-specific frequency was calculated using a Fisher's exact test. **f,** barplot as in **e** but for all GREs that are either hominin, human or chimpanzee-specific gain. **g,** heatmap showing the enrichment of disease associated GREs measured to be deregulated in autism spectrum disorder (ASD) and Alzheimer's disease (AD) patient brains in correlation to hominin-specific changes found in white matter (WM). Color coding indicates odds ratio of enrichment compared to all identified GRE. P-values were determined using a Fisher's exact test and represented in the heatmap if $p < 0.01$. **h,** metaplot analysis showing the average H3K27ac enrichment profile in different cell types for hominin-specific white matter gains. Source data are provided as a Source Data file.
