# Supplementary material for: Hominin-specific regulatory elements selectively emerged in oligodendrocytes and are disrupted in autism patients
Source: Nat Commun. 2020 Jan 16;11:301. doi: 10.1038/s41467-019-14269-w (PMC6965079; doi:10.1038/s41467-019-14269-w)
Supplement: Supplementary file 3 — Reporting Summary [file 41467_2019_14269_MOESM3_ESM.pdf]

nature research

Corresponding author(s): Menno Creyghton

Last updated by author(s): Dec 12, 2019

Reporting Summary

Nature Research wishes to improve the reproducibility of the work that we publish. This form provides structure for consistency and transparency in reporting. For further information on Nature Research policies, see Authors & Referees and the Editorial Policy Checklist .

Statistics

For all statistical analyses, confirm that the following items are present in the figure legend, table legend, main text, or Methods section.

n/a	Confirmed	
☐	☒	The exact sample size (*n*) for each experimental group/condition, given as a discrete number and unit of measurement
☐	☒	A statement on whether measurements were taken from distinct samples or whether the same sample was measured repeatedly
☐	☒	The statistical test(s) used AND whether they are one- or two-sided<br>*Only common tests should be described solely by name; describe more complex techniques in the Methods section.*
☐	☒	A description of all covariates tested
☐	☒	A description of any assumptions or corrections, such as tests of normality and adjustment for multiple comparisons
☐	☒	A full description of the statistical parameters including central tendency (e.g. means) or other basic estimates (e.g. regression coefficient) AND variation (e.g. standard deviation) or associated estimates of uncertainty (e.g. confidence intervals)
☐	☒	For null hypothesis testing, the test statistic (e.g. *F*, *t*, *r*) with confidence intervals, effect sizes, degrees of freedom and *P* value noted<br>*Give P values as exact values whenever suitable.*
☒	☐	For Bayesian analysis, information on the choice of priors and Markov chain Monte Carlo settings
☒	☐	For hierarchical and complex designs, identification of the appropriate level for tests and full reporting of outcomes
☐	☒	Estimates of effect sizes (e.g. Cohen's *d*, Pearson's *r*), indicating how they were calculated

Our web collection on statistics for biologists contains articles on many of the points above.

Software and code

Policy information about availability of computer code

Data collection	no software was used to collect the data.
Data analysis	Bowtie version 1.1.2 Langmead and Salzberg, 2012 http://bowtie-bio.sourceforge.net<br>Samtools version 1.3.1 Li, H. et al. 2009 http://samtools.sourceforge.net/<br>MACS2 version 2.1.1 Zhang, Y. et al. 2008 http://liulab.dfci.harvard.edu/MACS/<br>Bedtools version 2.26.0 Quinlan, A. R. & Hall, I. M. 2010 http://bedtools.readthedocs.io/en/latest/<br>R version 3.5.1 R Development Core Team, 2012 https://cran.r-project.org<br>IGV version 2.3.40 Robinson et al., 2011 http://software.broadinstitute.org/software/igv<br>t-SNE R package Van der Maarten et al. 2008 https://cran.r-project.org/web/packages/tsne/<br>DESeq2 R package Love, M.I. et al. 2014 https://bioconductor.org/packages/release/bioc/html/DESeq2.html<br>HISAT2 version 2.0.5 Kim, D. et al. 2015 https://ccb-jhu-edu.proxy.library.uu.nl/software/hisat2/manual.shtml<br>GREAT version 3.0.0 McLean, C.Y et al. 2010 http://bejerano.stanford.edu/great/public/html/<br>Homer version 4.9.1 Heinz, S. et al. 2010 http://homer.ucsd.edu/homer/index.html<br>Fastx-toolkit 0.0.13 http://hannonlab.cshl.edu/fastx_toolkit/index.html<br>cutadapt version 1.13 Martin M. 2010 https://cutadapt.readthedocs.io/en/stable/<br>ngs.plot version 2.47.1 Shen L. et al 2014 https://github.com/shenlab-sinai/ngsplot<br>LDSC version 1.0.1 Finucane H.K. 2015 https://github.com/bulik/ldsc<br>LLM3D Geeven G. et al. 2011

For manuscripts utilizing custom algorithms or software that are central to the research but not yet described in published literature, software must be made available to editors/reviewers. We strongly encourage code deposition in a community repository (e.g. GitHub). See the Nature Research guidelines for submitting code & software for further information.

Data

Policy information about availability of data

All manuscripts must include a data availability statement. This statement should provide the following information, where applicable:

- Accession codes, unique identifiers, or web links for publicly available datasets
- A list of figures that have associated raw data
- A description of any restrictions on data availability

- The ChIP-seq, RNA-Seq and ATAC-seq data reported in this study is available at Gene Expression Omnibus with accession code GSE130871
- Figures: 1b-d, 2b-g, 3a-d, 4b,d,e, S1a-d, S2a-f, S3a-d, S4a-c, S5a,b-c, S6a-g, S7a,d-j, S8c,d, S9c
- No restrictions on data availability
- reviewer token: clmvoowiflmpfkz

Field-specific reporting

Please select the one below that is the best fit for your research. If you are not sure, read the appropriate sections before making your selection.

☒ Life sciences ☐ Behavioural & social sciences ☐ Ecological, evolutionary & environmental sciences

For a reference copy of the document with all sections, see nature.com/documents/nr-reporting-summary-flat.pdf

Life sciences study design

All studies must disclose on these points even when the disclosure is negative.

Sample size	No sample size calculation was performed. As our sample represent tissues of rare availability all obtained specimens were used.
Data exclusions	For the DEseq2 analysis, we excluded all regions with zero reads within a single species. As we cannot determine whether this is due to the the inactivity or mappibility of the locus.
Replication	Observed H3K27ac enrichment was validated in publically availably ChIP-seq datasets.
Randomization	Primate species were assigned to a major evolutionary clades based on the reported phylogenetic tree.
Blinding	No blinding was done in this study.

Reporting for specific materials, systems and methods

We require information from authors about some types of materials, experimental systems and methods used in many studies. Here, indicate whether each material, system or method listed is relevant to your study. If you are not sure if a list item applies to your research, read the appropriate section before selecting a response.

Materials & experimental systems

n/a	Involved in the study	
☐	☒	Antibodies
☒	☐	Eukaryotic cell lines
☒	☐	Palaeontology
☒	☐	Animals and other organisms
☒	☐	Human research participants
☒	☐	Clinical data

Methods

n/a	Involved in the study	
☐	☒	ChIP-seq
☒	☐	Flow cytometry
☒	☐	MRI-based neuroimaging

Antibodies

Antibodies used	Rabbit polyclonal to Histone H3 (acetyl K27) - ChIP Grade, Abcam, #ab4729<br>Rabbit polyclonal to Histone H3 (tri methyl K4) - ChIP Grade, Abcam, #ab8580
Validation	Both antibodies (Abcam, ab4729 & Abcam ab8580) are frequently used in studies, including ENCODE (ENCODE Project Consortium, Nature 2012) and Roadmap Epigenomics (Bernstein et al. Nature Biotechnology 2010). Both are validated by members of the NIH epigenomics mapping consortium (Bernstein et al. Nature Biotechnology 2010).

ChIP-seq

Data deposition

- [x] Confirm that both raw and final processed data have been deposited in a public database such as GEO.
- [x] Confirm that you have deposited or provided access to graph files (e.g. BED files) for the called peaks.

Data access links
May remain private before publication.

GEO access GSE130871 https://www.ncbi.nlm.nih.gov/geo/query/acc.cgi?acc=GSE130871
reviewer token: clmvoowiflmpfkz

Files in database submission

Marmoset_Cerebellum_H3K27ac_1_calJac3.bw bigwig
Marmoset_Cerebellum_H3K27ac_2_calJac3.bw bigwig
Marmoset_Cerebellum_H3K27ac_3_calJac3.bw bigwig
Marmoset_Cerebellum_H3K4me3_1_calJac3.bw bigwig
Marmoset_Cerebellum_H3K4me3_2_calJac3.bw bigwig
Marmoset_PrefrontalCortex_H3K27ac_1_calJac3.bw bigwig
Marmoset_PrefrontalCortex_H3K27ac_2_calJac3.bw bigwig
Marmoset_PrefrontalCortex_H3K27ac_3_calJac3.bw bigwig
Marmoset_PrefrontalCortex_H3K4me3_1_calJac3.bw bigwig
Marmoset_PrefrontalCortex_H3K4me3_2_calJac3.bw bigwig
Human_Cerebellum_H3K27ac_2_hg38.bw bigwig
Human_WhiteMatter_H3K27ac_1.bw bigwig
Human_WhiteMatter_H3K27ac_2.bw bigwig
Human_WhiteMatter_H3K27ac_3.bw bigwig
Chimpanzee_WhiteMatter_H3K27ac_1.bw bigwig
Chimpanzee_WhiteMatter_H3K27ac_2.bw bigwig
Chimpanzee_WhiteMatter_H3K27ac_3.bw bigwig
Macaque_WhiteMatter_H3K27ac_1.bw bigwig
Macaque_WhiteMatter_H3K27ac_2.bw bigwig
Macaque_WhiteMatter_H3K27ac_3.bw bigwig
Marmoset_WhiteMatter_H3K27ac_1.bw bigwig
Marmoset_WhiteMatter_H3K27ac_2.bw bigwig
Marmoset_WhiteMatter_H3K27ac_3.bw bigwig
Human_PrefrontalCortex_NeuNnegative_ATAC_1.bw bigwig
Human_PrefrontalCortex_NeuNpositive_ATAC_1.bw bigwig
Human_PrefrontalCortex_NeuNnegative_ATAC_2.bw bigwig
Human_PrefrontalCortex_NeuNpositive_ATAC_2.bw bigwig
Chimpanzee_PrefrontalCortex_NeuNnegative_ATAC_4.bw bigwig
Chimpanzee_PrefrontalCortex_NeuNpositive_ATAC_4.bw bigwig
Chimpanzee_PrefrontalCortex_NeuNnegative_ATAC_5.bw bigwig
Chimpanzee_PrefrontalCortex_NeuNpositive_ATAC_5.bw bigwig
Macaque_PrefrontalCortex_NeuNnegative_ATAC_2.bw bigwig
Macaque_PrefrontalCortex_NeuNpositive_ATAC_2.bw bigwig
Macaque_PrefrontalCortex_NeuNnegative_ATAC_3.bw bigwig
Macaque_PrefrontalCortex_NeuNpositive_ATAC_3.bw bigwig
Marmoset_PrefrontalCortex_NeuNnegative_ATAC_1.bw bigwig
Marmoset_PrefrontalCortex_NeuNpositive_ATAC_1.bw bigwig
Marmoset_PrefrontalCortex_NeuNnegative_ATAC_2.bw bigwig
Marmoset_PrefrontalCortex_NeuNpositive_ATAC_2.bw bigwig
Marmoset_Cerebellum_H3K27ac_1_peaks.narrowPeak narrowPeaks
Marmoset_Cerebellum_H3K27ac_2_peaks.narrowPeak narrowPeaks
Marmoset_Cerebellum_H3K27ac_3_peaks.narrowPeak narrowPeaks
Marmoset_Cerebellum_H3K4me3_1_peaks.narrowPeak narrowPeaks
Marmoset_Cerebellum_H3K4me3_2_peaks.narrowPeak narrowPeaks
Marmoset_PrefrontalCortex_H3K27ac_1_peaks.narrowPeak narrowPeaks
Marmoset_PrefrontalCortex_H3K27ac_2_peaks.narrowPeak narrowPeaks
Marmoset_PrefrontalCortex_H3K27ac_3_peaks.narrowPeak narrowPeaks
Marmoset_PrefrontalCortex_H3K4me3_1_peaks.narrowPeak narrowPeaks
Marmoset_PrefrontalCortex_H3K4me3_2_peaks.narrowPeak narrowPeaks
Human_Cerebellum_H3K27ac_2_peaks.narrowPeak narrowPeaks
Human_WhiteMatter_H3K27ac_1.narrowPeak narrowPeaks
Human_WhiteMatter_H3K27ac_2.narrowPeak narrowPeaks
Human_WhiteMatter_H3K27ac_3.narrowPeak narrowPeaks
Chimpanzee_WhiteMatter_H3K27ac_1.narrowPeak narrowPeaks
Chimpanzee_WhiteMatter_H3K27ac_2.narrowPeak narrowPeaks
Chimpanzee_WhiteMatter_H3K27ac_3.narrowPeak narrowPeaks
Macaque_WhiteMatter_H3K27ac_1.narrowPeak narrowPeaks

Macaque_WhiteMatter_H3K27ac_2.narrowPeak narrowPeaks
Macaque_WhiteMatter_H3K27ac_3.narrowPeak narrowPeaks
Marmoset_WhiteMatter_H3K27ac_1.narrowPeak narrowPeaks
Marmoset_WhiteMatter_H3K27ac_2.narrowPeak narrowPeaks
Marmoset_WhiteMatter_H3K27ac_3.narrowPeak narrowPeaks
Human_PrefrontalCortex_NeuNnegative_ATAC_1.narrowPeak narrowPeaks
Human_PrefrontalCortex_NeuNpositive_ATAC_1.narrowPeak narrowPeaks
Human_PrefrontalCortex_NeuNnegative_ATAC_2.narrowPeak narrowPeaks
Human_PrefrontalCortex_NeuNpositive_ATAC_2.narrowPeak narrowPeaks
Chimpanzee_PrefrontalCortex_NeuNnegative_ATAC_4.narrowPeak narrowPeaks
Chimpanzee_PrefrontalCortex_NeuNpositive_ATAC_4.narrowPeak narrowPeaks
Chimpanzee_PrefrontalCortex_NeuNnegative_ATAC_5.narrowPeak narrowPeaks
Chimpanzee_PrefrontalCortex_NeuNpositive_ATAC_5.narrowPeak narrowPeaks
Macaque_PrefrontalCortex_NeuNnegative_ATAC_2.narrowPeak narrowPeaks
Macaque_PrefrontalCortex_NeuNpositive_ATAC_2.narrowPeak narrowPeaks
Macaque_PrefrontalCortex_NeuNnegative_ATAC_3.narrowPeak narrowPeaks
Macaque_PrefrontalCortex_NeuNpositive_ATAC_3.narrowPeak narrowPeaks
Marmoset_PrefrontalCortex_NeuNnegative_ATAC_1.narrowPeak narrowPeaks
Marmoset_PrefrontalCortex_NeuNpositive_ATAC_1.narrowPeak narrowPeaks
Marmoset_PrefrontalCortex_NeuNnegative_ATAC_2.narrowPeak narrowPeaks
Marmoset_PrefrontalCortex_NeuNpositive_ATAC_2.narrowPeak narrowPeaks
Human_WhiteMatter_1_RNAseq.txt RNA counts
Human_WhiteMatter_2_RNAseq.txt RNA counts
Chimpanzee_WhiteMatter_1_RNAseq.txt RNA counts
Chimpanzee_WhiteMatter_2_RNAseq.txt RNA counts
Marmoset_WhiteMatter_1_RNAseq.txt RNA counts
Marmoset_WhiteMatter_2_RNAseq.txt RNA counts
Marmoset_WhiteMatter_1_RNAseq.txt RNA counts
Marmoset_WhiteMatter_2_RNAseq.txt RNA counts
Marmoset_Cerebellum_H3K27ac_1.fastq.gz fastq
Marmoset_Cerebellum_H3K27ac_2.fastq.gz fastq
Marmoset_Cerebellum_H3K27ac_3.fastq.gz fastq
Marmoset_Cerebellum_H3K4me3_1.fastq.gz fastq
Marmoset_Cerebellum_H3K4me3_2.fastq.gz fastq
Marmoset_PrefrontalCortex_H3K27ac_1.fastq.gz fastq
Marmoset_PrefrontalCortex_H3K27ac_2.fastq.gz fastq
Marmoset_PrefrontalCortex_H3K27ac_3.fastq.gz fastq
Marmoset_PrefrontalCortex_H3K4me3_1.fastq.gz fastq
Marmoset_PrefrontalCortex_H3K4me3_2.fastq.gz fastq
Marmoset_Cerebellum_Input.fastq.gz fastq
Marmoset_PrefrontalCortex_Input.fastq.gz fastq
Human_Cerebellum_H3K27ac_2.fastq.gz fastq
Human_WhiteMatter_H3K27ac_1.fastq.gz fastq
Human_WhiteMatter_H3K27ac_2.fastq.gz fastq
Human_WhiteMatter_H3K27ac_3.fastq.gz fastq
Chimpanzee_WhiteMatter_H3K27ac_1.fastq.gz fastq
Chimpanzee_WhiteMatter_H3K27ac_2.fastq.gz fastq
Chimpanzee_WhiteMatter_H3K27ac_3.fastq.gz fastq
Macaque_WhiteMatter_H3K27ac_1.fastq.gz fastq
Macaque_WhiteMatter_H3K27ac_2.fastq.gz fastq
Macaque_WhiteMatter_H3K27ac_3.fastq.gz fastq
Marmoset_WhiteMatter_H3K27ac_1.fastq.gz fastq
Marmoset_WhiteMatter_H3K27ac_2.fastq.gz fastq
Marmoset_WhiteMatter_H3K27ac_3.fastq.gz fastq
Human_PrefrontalCortex_NeuNnegative_ATAC_1.fastq.gz fastq
Human_PrefrontalCortex_NeuNpositive_ATAC_1.fastq.gz fastq
Human_PrefrontalCortex_NeuNnegative_ATAC_2.fastq.gz fastq
Human_PrefrontalCortex_NeuNpositive_ATAC_2.fastq.gz fastq
Chimpanzee_PrefrontalCortex_NeuNnegative_ATAC_4.fastq.gz fastq
Chimpanzee_PrefrontalCortex_NeuNpositive_ATAC_4.fastq.gz fastq
Chimpanzee_PrefrontalCortex_NeuNnegative_ATAC_5.fastq.gz fastq
Chimpanzee_PrefrontalCortex_NeuNpositive_ATAC_5.fastq.gz fastq
Macaque_PrefrontalCortex_NeuNnegative_ATAC_2.fastq.gz fastq
Macaque_PrefrontalCortex_NeuNpositive_ATAC_2.fastq.gz fastq
Macaque_PrefrontalCortex_NeuNnegative_ATAC_3.fastq.gz fastq
Macaque_PrefrontalCortex_NeuNpositive_ATAC_3.fastq.gz fastq
Marmoset_PrefrontalCortex_NeuNnegative_ATAC_1.fastq.gz fastq
Marmoset_PrefrontalCortex_NeuNpositive_ATAC_1.fastq.gz fastq

	Marmoset_PrefrontalCortex_NeuNnegative_ATAC_2.fastq.gz fastq<br>Marmoset_PrefrontalCortex_NeuNpositive_ATAC_2.fastq.gz fastq
Genome browser session<br>(e.g. UCSC)	Bigwig files from all ChIP-seq and ATAC-seq samples can be downloaded from GEO and visualized in Integrative Genome Browser (IGV).<br>GEO access GSE130871 https://www.ncbi.nlm.nih.gov/geo/query/acc.cgi?acc=GSE130871

Methodology

Replicates	We have generated H3K27ac ChIP-seq data for cerebellum and prefrontal cortex of three marmoset species. Differentially enrichment analysis was done with 3 human sample versus 3 rhesus macaque or marmoset samples. For chimpanzee, differentially enrichment analysis was done with 2 chimpanzee samples versus 3 rhesus macaque or marmoset samples.

Sequencing depth

ChIP-sequencing (Supplmentary Table 3):
Total sequencing reads, Uniquely mapped reads, Fraction of reads in narrowPeaks, length of reads, SE/PE
Marmoset_Cerebellum_H3K27ac_1,15777644,10343094,20.38%,36,SE
Marmoset_Cerebellum_H3K27ac_2,14070187,11174561,11.83%,36,SE
Marmoset_Cerebellum_H3K27ac_3,33948738,26626784,22.82%,36,SE
Marmoset_PrefrontalCortex_H3K27ac_1,36178201,20260326,10.01%,36,SE
Marmoset_PrefrontalCortex_H3K27ac_2,37292103,29385809,11.44%,36,SE
Marmoset_PrefrontalCortex_H3K27ac_3,39702004,29972436,4.66%,36,SE
Marmoset_Cerebellum_H3K4me3_1,11709320,8819213,31.82%,36,SE
Marmoset_Cerebellum_H3K4me3_2,11559319,6549852,8.75%,36,SE
Marmoset_PrefrontalCortex_H3K4me3_1,10485374,7781895,27.82%,36,SE
Marmoset_PrefrontalCortex_H3K4me3_2,16317000,12550519,19.35%,36,SE
Marmoset_Cerebellum_Input,23580385,15664518,NA,36,SE
Marmoset_PrefrontalCortex_Input,13937158,9813827,NA,36,SE
Human_WhiteMatter_H3K27ac_1,24175260,16939091,1.62%,75,SE
Human_WhiteMatter_H3K27ac_2,29035691,20433617,2.74%,75,SE
Human_WhiteMatter_H3K27ac_3,33993601,23967317,4.80%,75,SE
Chimpanzee_WhiteMatter_H3K27ac_1,19312940,12817406,4.43%,75,SE
Chimpanzee_WhiteMatter_H3K27ac_2,32475765,22551091,5.80%,75,SE
Chimpanzee_WhiteMatter_H3K27ac_3,31771720,22187212,10.44%,75,SE
Macaque_WhiteMatter_H3K27ac_1,28674986,19287171,2.27%,75,SE
Macaque_WhiteMatter_H3K27ac_2,30919268,20450752,3.42%,75,SE
Macaque_WhiteMatter_H3K27ac_3,28126109,18576681,3.83%,75,SE
Marmoset_WhiteMatter_H3K27ac_1,30562459,21812606,7.98%,75,SE
Marmoset_WhiteMatter_H3K27ac_2,36960153,26191125,9.88%,75,SE
Marmoset_WhiteMatter_H3K27ac_3,30362933,21069387,10.21%,75,SE

RNA-seq (Supplementary Table 3):
Total sequencing reads, Uniquely mapped reads, length of reads, SE/PE
Human_WhiteMatter_RNAseq_1,28732038,3767451,60,SE
Human_WhiteMatter_RNAseq_2,30303680,6245283,60,SE
Chimpanzee_WhiteMatter_RNAseq_1,28127951,4769441,60,SE
Chimpanzee_WhiteMatter_RNAseq_2,24391066,2705467,60,SE
Macaque_WhiteMatter_RNAseq_1,25830619,6449453,60,SE
Macaque_WhiteMatter_RNAseq_2,31487620,8848574,60,SE
Marmoset_WhiteMatter_RNAseq_1,40821726,11877598,60,SE
Marmoset_WhiteMatter_RNAseq_2,32793030,12568247,60,SE

ATAC-seq (Supplementary Table 3):
Total sequencing reads, Uniquely mapped reads, length of reads, SE/PE
Human_PrefrontalCortex_NeuNnegative_ATAC_1,37184630,24752514,7.43%,75,SE
Human_PrefrontalCortex_NeuNpositive_ATAC_1,67188052,35797922,19.21%,75,SE
Human_PrefrontalCortex_NeuNnegative_ATAC_2,31182598,18312781,10.65%,75,SE
Human_PrefrontalCortex_NeuNpositive_ATAC_2,52090464,19037363,2.52%,75,SE
Chimpanzee_PrefrontalCortex_NeuNnegative_ATAC_4,38781601,22826404,18.66%,75,SE
Chimpanzee_PrefrontalCortex_NeuNpositive_ATAC_4,27450839,10708886,6.84%,75,SE
Chimpanzee_PrefrontalCortex_NeuNnegative_ATAC_5,41285772,19642867,14.16%,75,SE
Chimpanzee_PrefrontalCortex_NeuNpositive_ATAC_5,31190732,12059991,9.11%,75,SE
Macaque_PrefrontalCortex_NeuNnegative_ATAC_2,22572579,4773206,17.32%,75,SE
Macaque_PrefrontalCortex_NeuNpositive_ATAC_2,28217173,12091448,24.19%,75,SE
Macaque_PrefrontalCortex_NeuNnegative_ATAC_3,28151278,11809410,32.67%,75,SE
Macaque_PrefrontalCortex_NeuNpositive_ATAC_3,26071130,16795833,30.51%,75,SE
Marmoset_PrefrontalCortex_NeuNnegative_ATAC_1,30612892,16319082,6.86%,75,SE
Marmoset_PrefrontalCortex_NeuNpositive_ATAC_1,19006548,10349562,1.27%,75,SE
Marmoset_PrefrontalCortex_NeuNnegative_ATAC_2,21928305,4786839,3.83%,75,SE
Marmoset_PrefrontalCortex_NeuNpositive_ATAC_2,25673925,13881479,5.98%,75,SE

Antibodies

Rabbit polyclonal to Histone H3 (acetyl K27) - ChIP Grade, Abcam, #ab4729
Rabbit polyclonal to Histone H3 (tri methyl K4) - ChIP Grade, Abcam, #ab8580

Peak calling parameters

ChIP-seq reads were trimmed to a 36 bp length using the Fastx-toolkit (http://hannonlab.cshl.edu/fastx_toolkit/index.html) while for ATAC-seq reads the Nextera adapter sequences (5'-CTGTCTCTTATA-3') was trimmed using cutadapt (https://cutadapt.readthedocs.io/en/stable/). Sequence reads were aligned using Bowtie 1.1.2 (http://bowtie-bio.sourceforge.net/index.shtml), excluding reads with more than one mismatch (seed length 36) or with multiple alignments. Statistical significant H3K27ac or ATAC enriched regions compared to background were calculated for each sample using MACS2 version 2.1.1 (pvalue < 10-5, extsize 300, local lambda 100,000). Regions were extended to a minimum of 2000bp in length (+/- 1000bp from peak center) which reflects the typical resolution of H3K27ac enrichment. Duplicate reads were removed from the bam files using Samtools 1.3.1 and read coverage within enriched regions was counted using Bedtools v2.26.0

Data quality	Percentage of aligned and unique reads was higher than 70% for all samples. Fraction of reads in peaks per sample exceeded the 1% threshold used by the Encyclopedia of DNA Elements (ENCODE) (Supplementary Table 3)
Software	Bowtie version 1.1.1 Langmead and Salzberg, 2012 http://bowtie-bio.sourceforge.net<br>Samtools version 1.5 Li, H. et al. 2009 http://samtools.sourceforge.net/<br>MACS2 version 2.1.1 Zhang, Y. et al. 2008 http://liulab.dfci.harvard.edu/MACS/<br>Bedtools version 2.26.0 Quinlan, A. R. & Hall, I. M. 2010 http://bedtools.readthedocs.io/en/latest/<br>R version 3.5.1 R Development Core Team, 2012 https://cran.r-project.org<br>T-SNE R package Van der Maarten et al. 2008 https://cran.r-project.org/web/packages/tsne/<br>DESeq2 R package Love, M.I. et al. 2014 https://bioconductor.org/packages/release/bioc/html/DESeq2.html<br>Fastx-toolkit 0.0.13 http://hannonlab.cshl.edu/fastx_toolkit/index.html<br>cutadapt version 1.13 Martin M. 2010 https://cutadapt.readthedocs.io/en/stable/
