# Supplementary material for: Hominin-specific regulatory elements selectively emerged in oligodendrocytes and are disrupted in autism patients
Source: Nat Commun. 2020 Jan 16;11:301. doi: 10.1038/s41467-019-14269-w (PMC6965079; doi:10.1038/s41467-019-14269-w)
Supplement: Supplementary file 4 — Description of Additional Supplementary Files [file 41467_2019_14269_MOESM4_ESM.docx]

**Description of Additional Supplementary Files**

Supplementary Data 1

Description: Table containing information about the common marmosets used for samples.

Supplementary Data 2

Description: Table containing all the accession codes and references to the publicly available data sets used in this study.

Supplementary Data 3

Description: Sequencing statistics of all newly generated ChIP-sequencing and RNA-sequencing data sets.

Supplementary Data 4

Description: Non-redundant list of primate regulatory elements used in this study. Regions were reciprocally mapped across the four primate genomes and only those were retained that could be mapped on all genomes.

Supplementary Data 5

Description: Table containing the number of regulatory elements per genetic location on the human genome. For all regions as well as those annotated as hominin-specific gain or loss.

Supplementary Data 6

Description: Lists of regulatory elements that were identified as hominin-gain and hominin-loss for both cerebellum as well as prefrontal cortex.

Supplementary Data 7

Description: Motif-analysis using HOMER on hominin-specific regulatory changes of both cerebellum as well as prefrontal cortex.

Supplementary Data 8

Description: Gene-onthology using GREAT on hominin-specific regulatory changes of both cerebellum as well as prefrontal cortex.

Supplementary Data 9

Description: List of hominin-specific prefrontal cortex gains that overlap regions lost specifically in the prefrontal cortex of autism patients.
